# A Novel Citrullinated Modification of Histone 3 and Its Regulatory Mechanisms Related to IPO-38 Antibody-Labeled Protein

**DOI:** 10.3389/fonc.2019.00304

**Published:** 2019-04-18

**Authors:** Shuzheng Song, Zhen Xiang, Jun Li, Jun Ji, Ranlin Yan, Zhenggang Zhu, Yingyan Yu

**Affiliations:** Department of Surgery, Ruijin Hospital affiliated to Shanghai Jiao Tong University School of Medicine, Shanghai Key Laboratory for Gastric Neoplasms, Shanghai, China

**Keywords:** IPO-38, histone modification, citrullination, PADI4, biomarker

## Abstract

IPO-38 is a potential biomarker for early diagnosis of gastric cancer that we recently identified. Although we characterized its chemical nature as a nucleosome histone, we suspected the existence of histone modification for the IPO-38 antibody-labeled protein. Here, we used a commercially available modified histone peptide array to identify the type and site of histone modification labeled by the IPO-38 monoclonal antibody. In protein array analysis, the citrulline modification of histone 3 on arginine 26 (H3R26Cit) yielded the strongest signal. Although peptidyl arginine deiminase-2 and -4 (PADI2 and PADI4, respectively) can catalyze the conversion of arginine to citrulline, we observed that only PADI4 expression correlated with the citrulline histone modification of H3R26Cit. Overexpression of PADI4, via transfection of a eukaryotic expression vector, and knockdown of PADI4 gene expression, by a PADI4 CRISPR/Cas9 vector, confirmed the crucial function of PADI4 on the increased level of H3R26Cit in gastric cancer cell lines. By immunoprecipitation and immunoblotting, we found an interaction between H3R26Cit and H3K27me3. Our study established the first link between the IPO-38 antigen and citrullinated histone 3, and clarified the upstream regulatory enzyme PADI4. The new findings suggest an important role for the citrullination modification of histone in gastric cancer biology, and should help us optimize the development of a sensitive and specific diagnostic reagent.

## Introduction

Gastric cancer is a disease with high morbidity and mortality rates worldwide, especially in East Asia. Data from GLOBOCAN 2018 show there are 1,033,701 new cases and 782,685 death cases of gastric cancer all over the world (1). Currently, methods are limited for early diagnosis of gastric cancer. Patients are often diagnosed with gastric cancer at an advanced stage with poor prognosis. Therefore, early diagnosis is a key to improving the outcome of patients. Our group proposed a candidate biomarker IPO-38 for diagnosis of gastric cancer (2). Assaying IPO-38 provides significantly higher specificity and sensitivity (56.7 and 93.3%, respectively), over routinely used biomarkers CEA, CA199, and CA72-4. IPO-38 has long been used as a cell proliferation nuclear antigen (3, 4). Although we identified it as a member of the histone protein family based on mass spectrometry, we considered that the histone was modified chemically (2).

Protein function is specified by appropriately folded secondary structure and post-translational modifications, including acetylation, methylation, phosphorylation, and citrullination (5). Histone modification plays an important role in maintaining homeostasis. Disorders of histone modification associate with cancer, neurological diseases, as well as autoimmune diseases (6, 7). Histone modifications potentially alter the electrical charge between histones and DNA duplexes, impacting chromatin organization and transcription. Histone modifications also affect gene regulation by modulating binding with transcription factors (6–8). In addition, histone modifications are involved in the formation of neutrophil extracellular traps (NETs), a crucial process for microbe clearance (9), which also plays a role on cancer metastasis through protein citrullination in peripheral blood (10–12).

Specific antibody analysis and mass spectrometry are commonly used for detection of histone modifications. However, the number of histone-specific antibodies is limited, which has restricted progress in studying histone modifications and functions. Mass spectrometry potentially overcomes the defect of insufficient antibodies to some extent, but trypsin digestion in the sample pretreatment step often destroys many modification sites, and ultimately reduces sensitivity (13). In 2010, a new histone modified peptide array was developed, promoting research to understand the function, metabolism, and significance of histone modifications (14–16).

To clarify the histone modification characteristics and biological significance of the IPO-38 antigen, we used the modified peptide array to identify the IPO-38 monoclonal antibody-binding protein. We characterized the novel modified histone H3, and identified that PADI4 is a key enzyme catalyzing citrullination modification of histone 3.

## Materials and Methods

### Modified Histone Peptide Array Analysis

MODified™ Histone Peptide Array from Active motif (Active Motif, California, USA) is a histone modified polypeptide chip of 59 single-site histone modifications and different permutations in 384 dot matrixes. Each chip is divided into left and right wings and repeating lattice arrangement. The chip was first blocked with 5% BSA (Sangon Biotech, Shanghai, China) for 1 h at room temperature, and then incubated with IPO-38 monoclonal antibody (1:1000, Thermo Fisher, Massachusetts, USA) overnight at 4°C. The next day, the chip was washed three times with 1 × PBST [1 × PBS with 0.1% (v/v) Tween-20], and then incubated with HRP-labeled goat anti-mouse IgM second antibody for 1 h at room temperature (1:5000, Sangon Biotech, Shanghai, China). After incubation, the chip was again washed with 1 × PBST three times and the signal was detected using ECL luminescent reagent (Meilun, Shanghai China), in a chemiluminometer (Tanon, Shanghai, China). The histone modification sites and signal intensity analysis were conducted with the special software provided by Active Motif (https://www.activemotif.com/catalog/668).

### Cell Culture

Gastric cancer cell lines, SGC7901, MKN45, HGC27, and BGC823, were purchased from the Cell Bank of the Chinese Academy of Sciences (Shanghai, China). Gastric cancer cell lines, Hs746T, AGS, and NCI-N87, were purchased from the American Type Culture Collection (ATCC, Maryland, USA), and the human gastric mucosal cells, GES1, and 293T cells were preserved in our laboratory. Cell lines were cultured in 37°C culture incubator with 5% carbon dioxide using RPMI 1640 or DMEM medium (Hyclone, Utah, USA) containing 10% FBS (Gibco, New York, USA) according to the manufacturer's instructions.

### Construction of PADI2 and PADI4 Eukaryotic Expression Vectors and PADI4 CRISPR/Cas9 Vector

Primers were designed for the coding region sequences of the PADI2 (NM_007365.2), PADI4 (NM_012387.2), and the restriction sites for the eukaryotic expression vector pCDH-CMV-MCS-EF1-Puro (SBI, California, USA). The high-fidelity PCR enzyme KOD plus neo (Toyobo, Osaka, Japan) was used to amplify the coding region sequences of PADI2 and PADI4 from a 293T cell cDNA library. Agarose gel (1%) electrophoresis was used to confirm the PCR product size, and T4 ligase (NEB, Massachusetts, USA) was used to link the target fragment to the empty linear vector after digestion. Competent TNF5α cells (Tiangen, Shanghai, China) were transformed with the expression vectors, and three positive colonies were selected for sequencing to verify the plasmid.

CRISPR/Cas9 vector targeting PADI4 (NM_012387.2) was constructed using the lentiCRISPRv2 vector, which was a gift from the Feng Zhang lab at MIT. The online guide RNA design website (http://crispr.mit.edu) was used to design the target sequence near the transcription start site of PADI4. The top two scored sequences were selected as the gene editing sites for primers (gRNA1: 5′-GGGACGAGCTAGCCCGACGA-3′; gRNA2: 5′-TCACACGGATCAATGTCCCC-3′). In this study we adopted an all-in-one method. Primers designed according to the two gRNA sequences and the tracRNA-U6 vector sequences were used to produce a gRNA1-tracRNA-U6-gRNA2 fragment. Then the proper fragment was ligated into the lentiCRISPRv2 vector and verified by sequencing.

### Lentiviral Packaging and Stable Cell Line Screening

The constructed eukaryotic expression vector and gene knockdown vector were transfected into the 293T cells with the packaging plasmids psPAX2 and pMD2.G using Lipofectamine 2000 (Thermo Fisher, Massachusetts, USA). The lentivirus was harvested 48 h after transfection, and the lentivirus supernatant was filtered using a 0.45 μm filter. One day prior to infection, the three cell lines (AGS, SGC7901, and MKN45) were plated at 2 × 10^5^ cells per well in 6-well tissue culture plates. The lentivirus was added into the separate cell lines, and polybrene was added at a density of 6 ng/ul (Sigma, California, USA). After 24 h, the infection medium was removed and replaced with normal culture medium. After 48 h, the cell lines were screened using 2 ng/μl puromycin (Sangon Biotech, Shanghai, China), and a stable cell line was formed after 1 week of continuous selection.

### Western Blot

Whole cellular protein was extracted using RIPA lysis buffer (Beyotime, Shanghai, China) containing a protease inhibitor cocktail (Roche, Basel, Switzerland). The cytoplasmic and nuclear protein fractions were isolated using a Nuclear and Cytoplasmic Protein Extraction Kit (Beyotime, Shanghai, China) according to the manufacturer's instructions. Protein samples were separated by SDS-PAGE gel containing 10% acrylamide, electrophoresis and transferred to a 0.45 μm PVDF membrane (Millipore, Massachusetts, USA). The transferred membranes were blocked with 5% BSA for 1 h at room temperature. Then the membranes were incubated with the corresponding primary antibodies: mouse anti-human IPO-38 monoclonal antibody (1:1000, Thermo Fisher, Massachusetts, USA); rabbit anti-human H3K27ac polyclonal antibody, rabbit anti-human H3R26Cit/H3K27me3 monoclonal antibody, and mouse anti-human PADI4 monoclonal antibody (1:1000, Abcam, Cambridge, UK); rabbit anti-human EZH2 monoclonal antibody (1:1000, CST, Boston, Massachusetts USA), rabbit anti-human PADI2 polyclonal antibody, (1:1000, Proteintech, Chicago, Illinois, USA), and HRP-labeled mouse anti-human GAPDH monoclonal antibody (1:2000, Proteintech, Chicago, Illinois, USA), and mouse anti-human histone H3 monoclonal antibody (1:1000, Abcam, Cambridge, UK) as an internal reference antibody overnight at 4°C. The next day, 1 × TBST buffer [10 mM Tris-HCl, pH 8.0, 150 mM NaCl, 0.1% (v/v) Tween-20] was used to wash the membranes 3 times for 10 min each time at RT. HRP-labeled goat anti-rabbit or mouse IgG secondary antibody (1:5000, Proteintech, Chicago, Illinois, USA) of the corresponding species was incubated for 1 h at RT. HRP-labeled goat anti-mouse IgM secondary antibody (Sangon Biotech, Shanghai, China) was used as the second antibody for the IPO-38 IgM monoclonal antibody. After the incubation of the secondary antibody, the membranes were washed 3 times for 10 min each time at RT with 1 × TBST buffer, and then the signal was detected in the chemiluminometer using ECL luminescent solution (Meilun, Shanghai China).

### Histone Immunoprecipitation

To reduce the interference of non-histone proteins and nucleotides, we used enzymatic digestion to obtain histones for further immunoprecipitation. After collecting the cell pellet, we used the hypotonic buffer [0.3 M sucrose, 60 mM KCl, 15 mM NaCl, 5 mM MgCl_2_, 0.1 mM EGTA, 15 mM Tris-HCl pH 7.5, 5 mM sodium butyrate, 0.4% NP40, and Complete™ EDTA-free protease inhibitor mixture (Roche, Basel, Switzerland)] to rupture the cell membrane, and then collected the nuclear pellet. Nuclear deposition concentration was measured by nanodrop (Thermo Fisher, Massachusetts, USA), and 200 U/5 μg of micrococcal nuclease (NEB, Massachusetts, USA) was used to digest the nucleosome at 37°C for 6 min. EDTA (Sigma, California, USA) was added to stop the reaction. After centrifugation, the supernatant, which contains histone DNA complexes, namely nucleosomes, was collected, and concentration was measured. The appropriate amount of lysate was taken as input, and the remainder was divided into 3 groups, and 20 μl of protein A/G magnetic beads (Thermo Fisher, Massachusetts, United States), and 5 μg of anti-H3K27me3 antibody, anti-H3K27ac antibody, or normal rabbit IgG (CST, Boston, Massachusetts, USA) was added to each sample of lysate. After incubating overnight at 4°C on a shaker, the complexes were washed with RIPA buffer three times in the magnetic frame (Invitrogen, California, USA). Finally, the bound proteins were eluted into 1 × SDS loading buffer (Beyotime, Shanghai, China). The subsequent steps followed the immunoblotting protocol described above, and the rabbit anti-human H3R26Cit polyclonal antibody was used to detect the corresponding histone modification.

### Immunofluorescence

The MKN45 and SGC7901 cancer cell lines (5 × 10^3^ cells per plate) were seeded on a fluorescence chamber culture plate. After the cells fully stretched and adhered to the plate 12 h later, they were fixed in 4% paraformaldehyde for 15 min at RT, and the cell and nuclear membranes were permeabilized in 0.5% Triton X-100 (Sangon Biotech, Shanghai, China) for 20 min at RT. The plate was washed 3 times for 5 min with 1 × PBS. Goat serum (Sangon Biotech, Shanghai, China) was used for antigen blocking for 1 h at RT. After blocking, the samples were incubated with mouse anti-human PADI4 monoclonal antibody (1:100) and rabbit anti-human PADI2 polyclonal antibody (1:100) at 4°C overnight in a wet box. The plate was then washed with 1 × PBST three times for 5 min each, and incubated with Alexa Fluor 488 goat anti-mouse red fluorescent secondary antibody and Alexa Fluor 555 goat anti-rabbit green fluorescent secondary antibody (1:250, Invitrogen, California, USA) at RT in the dark for 1 h. Nuclei were stained for 5 min at room temperature in the dark with DAPI (Sigma, California, USA). Finally, plates were washed 3 times for 5 min with 1 × PBST. Fluorescence signal could be observed and the fluorescent images were taken with a fluorescence microscope (Nikon, Tokyo, Japan).

### Real-Time PCR

Total mRNA was extracted from the cell lines using Trizol (Invitrogen, California, USA), according to the manufacturer's protocol. The obtained mRNA was reverse transcribed using the ReverTra Ace® qPCR RT Kit (Toyobo, Osaka, Japan). The mRNA levels of PADI2, PADI4, EZH2, KDM6A, KDM6B, and GAPDH were detected using the following specific primers: Primers for PADI2, forward: 5′- GCACCTACCTCTGGACCGAT-3′, reverse: 5′-ACACGTGTTCCGAGTGCTTC-3′, product length 81 bp; primers for PADI4, forward: 5′- GACCCCCAAGGACTTCTTCA-3′, reverse: 5′-GCTGCACTTGGAGGACAGTT-3′, product length 115 bp; primers for EZH2, forward: 5′-CATACGCTTTTCTGTAGGCGA-3′, reverse: 5′-TCCGCTTATAAGTGTTGGGTG-3′, product length 82 bp; primers for KDM6A, forward: 5′-TCTCCAAAAGTCCTTGGAAGC-3′, reverse: 5′-AAGGCATCCTGAACTTTCCC-3′, product length 96 bp; primers for KDM6B, forward: 5′-TACAGACCCTCGAAATCCCA-3′, reverse: 5′-CAGGGTCTTGGTGGAGAAGA-3′, product length 88 bp; and primers for GAPDH, forward: 5′-ACGGATTTGGTCGTATTGGGCG-3′, reverse: 5′-CTCCTGGAAGATGGTGATGG-3′, product length 212 bp. The qPCR reaction was carried out in a Roche Light cycler 480 PCR machine (Roche, Basel, Switzerland) using SYBR Green PCR master mix (Life Technologies, California, USA).

### Statistical Analysis

The mRNA expression data analysis was performed by Student's *t*-test using GraphPad Prism 8.0.1 software (GraphPad Software, San Diego, California, USA). Differences were considered statistically significant when *P* < 0.05.

## Results

### Identification of Histone Modifications Marked by the IPO-38 Monoclonal Antibody

**Figure 1:**
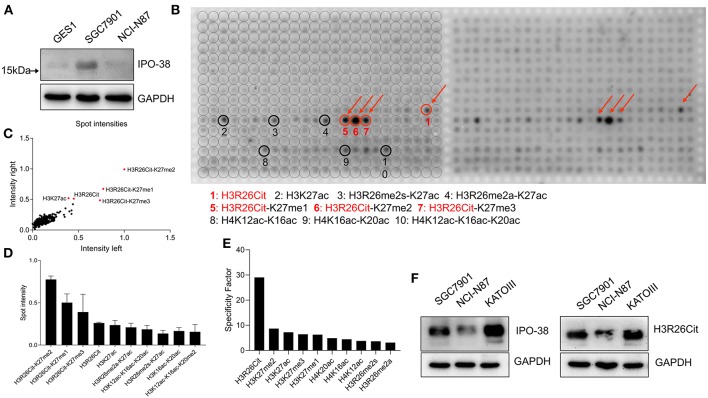
Analysis of histone modification peptide array using IPO-38 monoclonal antibody. **(A)** The protein expression of IPO-38 in gastric mucosal cell GES-1 and gastric cancer cell lines. **(B)** Presentation of signal intensity on modified histone peptide array based on incubation with the IPO-38 monoclonal antibody. **(C)** The consistency assay of two repeated detections on the modified histone peptide array. **(D)** The top 10 histone modifications with the strongest binding to the IPO-38 monoclonal antibody. **(E)** The top 10 histone modification sites with the best specificity for IPO-38 monoclonal antibody binding. **(F)** Comparison of H3R26Cit and IPO-38 protein levels in three gastric cancer cell lines.

The IPO-38 monoclonal antibody detects proteins with a molecular weight around 15 kDa in total cellular protein lysates of human gastric epithelial cells (GES1) and gastric cancer cell lines (SGC7901 and NCI-N87) (Figure 1A). After incubating the modified histone peptide chip with the IPO-38 monoclonal antibody, 10 high intensity signals were obtained that corresponded to: H3R26Cit-K27me2, H3R26Cit-K27me1, H3R26Cit-K27me3, H3R26Cit, H3K27ac, H3R26me2a-K27ac, H3K12ac-K16ac-K20ac, H3R26me2s-K27ac, H3K16ac-K20ac, and H3K12ac-K16ac-K20me2 (Figures 1B–D). Results were duplicated on the left and right wings of the chip (Figure 1B), and signal intensities aligned well and showed good consistency (Figure 1C). Specific analysis of modified histone peptides revealed that the highest specificity of IPO-38 antibody-binding was for H3R26Cit, followed by the H3K27me2 modification (Figure 1E). We noticed that the signal intensity of H3R26Cit site was significantly enhanced when the adjacent site H3K27 was methylated. In particular, the presence of K27me2 modification resulted in 3-fold up-regulation of signaling intensity than that of R26Cit alone based on signaling intensity analysis. Immunoblotting using an antibody specific for H3R26Cit correlated well with protein levels detected using the IPO-38 antibody in the gastric cancer cell lysates (Figure 1F).

### Expression Levels of H3R26Cit and Related Catalytic Enzyme PADIs

**Figure 2:**
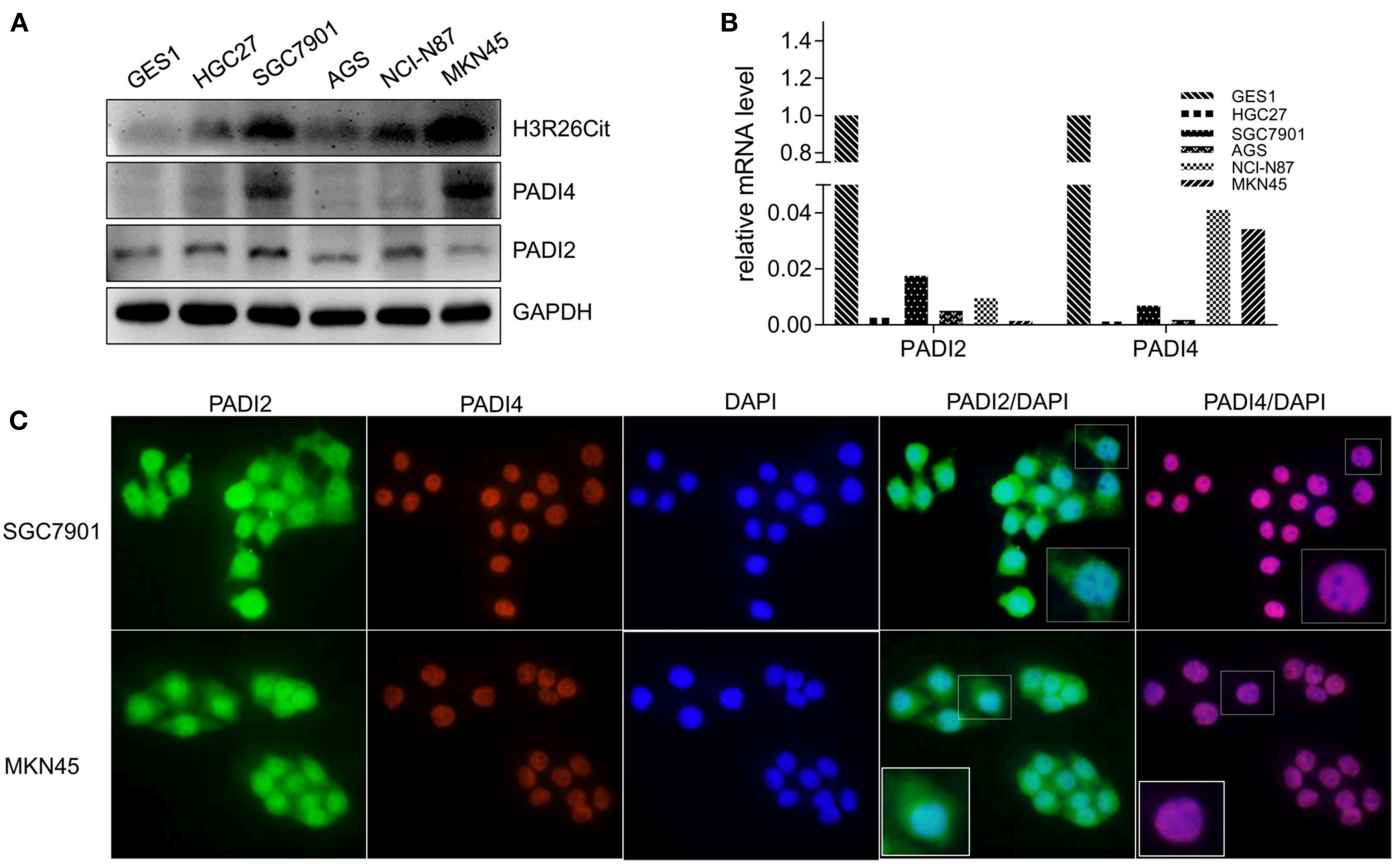
Analysis of basal expression of H3R26Cit and its catalytic enzymes PADIs. **(A)** The protein expression of H3R26Cit, PADI2, and PADI4 in GES-1 gastric mucosa cells and several gastric cancer cell lines. **(B)** The expression of PADI2 and PADI4 mRNA in GES-1 gastric mucosa cells and several gastric cancer cell lines. **(C)** Subcellular localization of PADI2 and PADI4 proteins in SGC7901 and MKN45 gastric cancer cell lines.

Since PADI2 or PADI4 catalyzes the conversion of arginine to citrulline in humans, we examined the protein levels of PADI2, PADI4, and H3R26Cit in several human gastric cancer cell lines. We observed that the basal expression level of H3R26Cit was higher in SGC7901 and MKN45 cells, and basal expression of PADI4 was also higher in those cancer cell lines. No significant difference of PADI2 was found in those cancer cell lines (Figure 2A). The mRNA expression level of PADI2 and PADI4 was lower in cancer cell lines, compared to GES1 control cells, by q-RT-PCR (Figure 2B), though PADI4 protein levels were higher in SGC7901 and MKN45 cells. There was discrepancy between the mRNA and protein levels of PADI2 and PADI4. By immunofluorescence microscopy, PADI2 was shown to localize in both the cytoplasm and nucleus, whereas PADI4 was found only in the nucleus (Figure 2C).

### The Impact of PADI2 and PADI4 Overexpression and Knockdown on H3R26Cit Level

**Figure 3:**
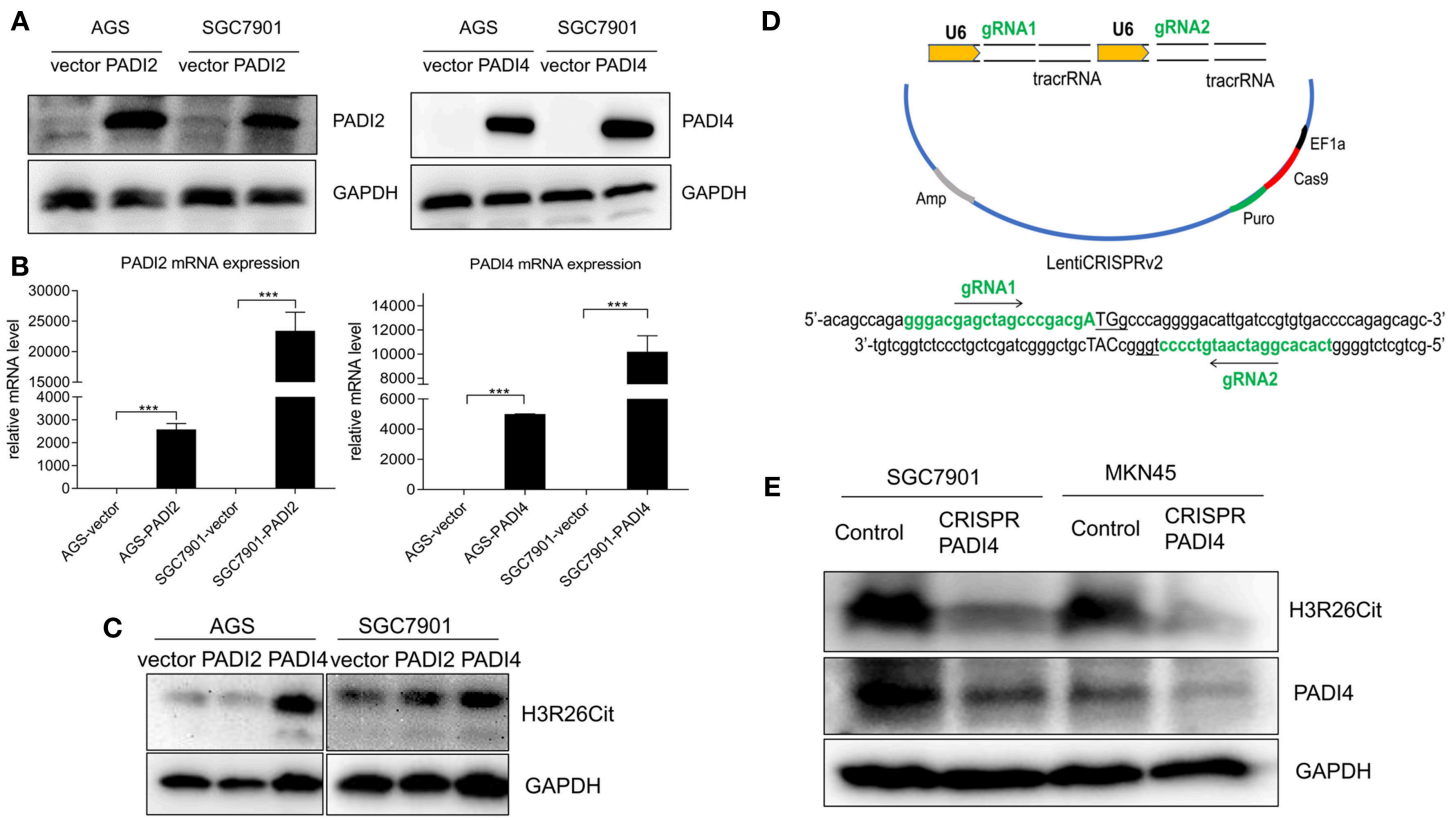
The influence of PADI2 and PADI4 overexpression or knockdown on H3R26Cit. **(A)** Detection of protein level changes after overexpression of PADI2 and PADI4. **(B)** Detection of mRNA level changes after overexpression of PADI2 and PADI4 (*** indicates *P* < 0.001). **(C)** The protein level of H3R26Cit is significantly increased after enforcing PADI4 expression, compared to enforcing PADI2 expression. **(D)** Schematic diagram of the construction of CRISPR/Cas9 all-in-one plasmid system with a double target on the PADI4 gene. **(E)** The protein level of H3R26Cit is significantly decreased after knockdown of PADI4 in both SGC7901 and MKN45 gastric cancer cell lines.

PADI2 and PADI4 eukaryotic expression vectors were packaged with lentivirus. Although PADI4 protein level was higher in SGC7901 and MKN45 cell lines (Figure 2A), but they took longer exposure time with ECL luminescence reagent (2 min). Then we chose a PADI4 low expression AGS cell line and a PADI4 moderate expression SGC7901 cell line for the overexpression study, and SGC7901 and MKN-45 cells were used for the knockdown study. After PADI2 and PADI4 were successfully expressed, we examined the expression level of H3R26Cit (Figures 3A,B). Overexpression of PADI4 significantly increased intracellular expression of H3R26Cit, compared to PADI2 overexpression, shown by both Western blot with shorter exposure time (2 s) (Figure 3C).

The “all-in-one” single plasmid dual target PADI4 gene knockdown system was constructed using CRISPR/Cas9 technology, which targeted a dual target near the PADI4 transcription start site (Figure 3D). The plasmid was packaged with lentivirus and SGC7901 and MKN45 cell lines were infected. The puromycin was used to select a stable cell line. The significant decrease in the expression level of PADI4 in experimental cells was accompanied by a decrease in the expression level of H3R26Cit (Figure 3E).

### Analysis of Interaction Between H3R26Cit and Other Post-translational Modification

**Figure 4:**
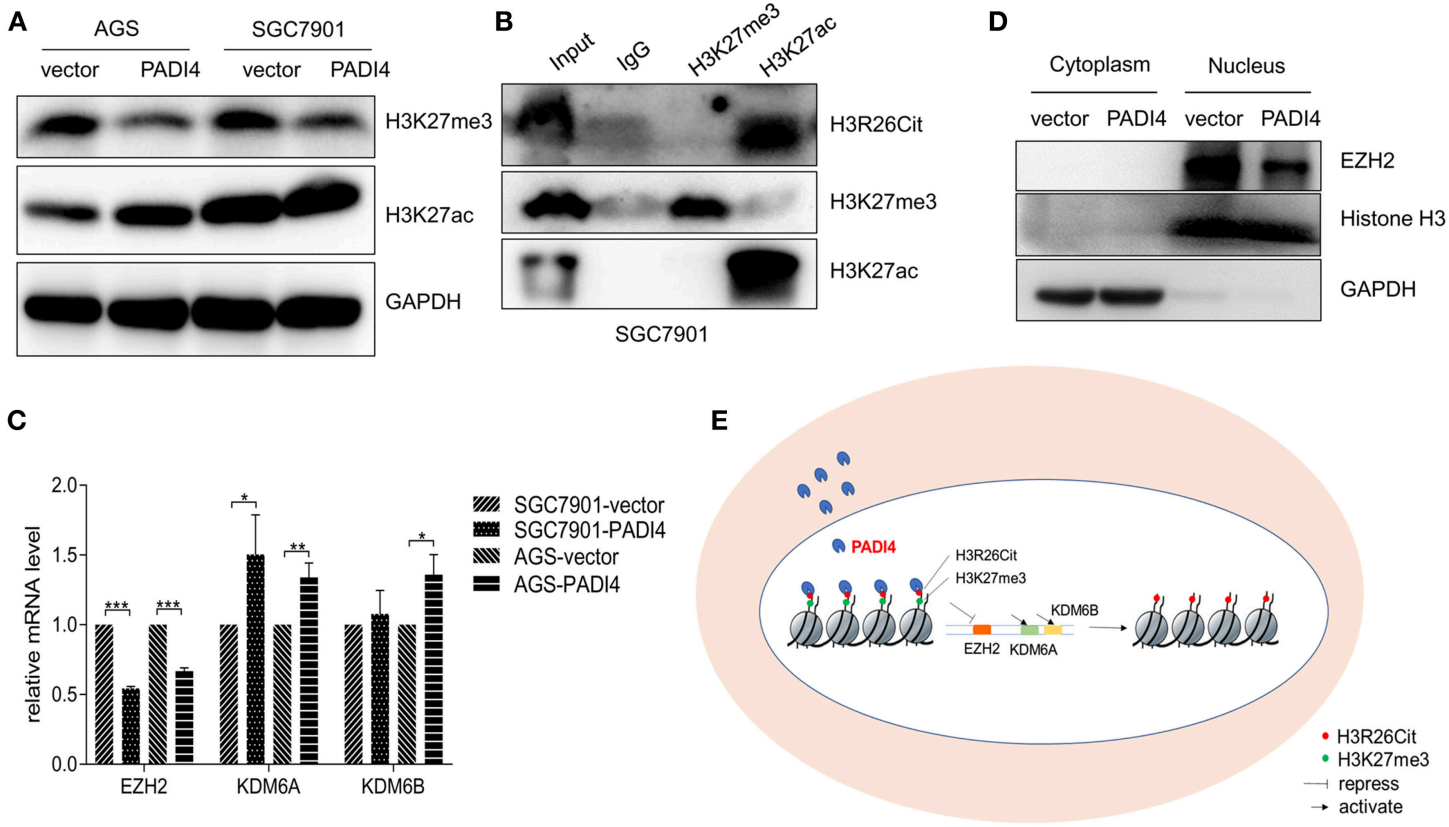
Interaction assay of H3R26Cit and other post-translational modifications. **(A)** An increase in the level of H3K27ac protein and decreased H3K27me3 protein level were observed in both AGS and SGC7901 gastric cancer cell lines in which PADI4 was overexpressed. **(B)** Immunoprecipitation was performed by H3K27me3 and H3K27ac antibodies. H3R26Cit was not detected in the H3K27me3 pull-down product, but was found in the H3K27ac pull-down product. **(C)** Effect of PADI4 overexpression on mRNA expression of the H3K27 methyltransferase EZH2 and demethylases, KDM6A and KDM6B (*, **, and *** represent *P* < 0.05, *P* < 0.01, and *P* < 0.001, respectively) **(D)** EZH2 expression assay revealed that the protein was located in nucleus, and its expression level was decreased after PADI4 overexpression, with histone 3 and GAPDH serving as internal controls. **(E)** Schematic diagram of influences on histone modifications of H3R26Cit and H3K27me3 after PADI4 overexpression.

Since H3K27ac and H3K27me3 were also highlighted in the modified histone peptide array, we analyzed the interaction between H3R26Cit and other histone modifications. As shown in Figure 4A, overexpression of PADI4 resulted in a significant decrease of H3K27me3 levels in AGS and SGC7901 cells, but led to increased expression of H3K27ac. To clarify the potential crosstalk between H3R26Cit and H3K27me, we extracted nucleosomes from cell nucleus by means of the micrococcal nuclease method, and performed immunoprecipitations using H3K27me3 and H3K27ac antibodies. H3R26Cit was not detected in the H3K27me3 pull-down product, but co-precipitated with H3K27ac (Figure 4B), which supports an interaction between H3R26Cit and H3K27ac. We further examined expression levels of EZH2, an H3K27me3 methyltransferase, and KDM6A/KDM6B demethylases after PADI4 overexpression. The expression level of EZH2 was significantly decreased in SGC7901 and AGS cells (*P* < 0.001), while the expression level of KDM6A was significantly increased (*P* = 0.037; *P* = 0.0046, for SGC7901 and AGS cells, respectively). The expression level of KDM6B was increased to some extent (*P* = 0.46; *P* = 0.012) (Figure 4C). A significant down-regulation of EZH2 in the nucleus was found; as internal controls, GAPDH was only expressed in the cytoplasm and histone 3 was only expressed in nucleus (Figure 4D). The results suggest that PADI4 not only catalyzes H3R26Cit modification, but also influences the activities of EZH2, KDM6A, and KDM6B, as reflected in the decreased level of H3K27me3 in the nucleus (Figure 4E).

## Discussion

IPO-38 is a diagnostic biomarker for gastric cancer identified in our previous clinical proteome study. We proposed that the protein labeled by IPO-38 monoclonal antibody was a nucleosome histone and suspected it was a modified histone H2B (2). We could not, however, clarify the exact histone modification due to insufficient methods.

In recent years, the relationship between histone modification and tumorigenesis has attracted greater attention. Technologies for detecting and studying histone modifications have been developed and greatly improved. Using the self-developed chromatin immunoprecipitation-based microarray method (ChIP-chip) technology, Heintzman and coworkers demonstrated that cell-specific histone modifications bound to cell-specific enhancers affect cell-specific gene expression spectrum (17). Cejas et al. developed fixed-tissue chromatin immunoprecipitation sequencing, which enables reliable extraction of soluble chromatin from formalin-fixed paraffin-embedded tissues for accurate detection of histone marks. By using multiple histone marks, they generated chromatin state maps and identified cis-regulatory elements in clinical samples for various tumor types (18).

In the current study, a modified histone peptides array was used. This protein array covers 59 different combinations of post-translational modifications such as methylation, acetylation, phosphorylation, and citrullination in up to four different modifications per peptide (15, 16). This array is suitable for assessing the specificity of histone-modified antibodies and for analyzing interactions between different histone modification sites. The processing is straightforward, similar to Western blotting, and used in different molecular oncology laboratories (15, 16, 19). By means of this protein array, we characterized the antigen labeled by the IPO-38 antibody as H3R26Cit, which could interact with H3K27me and form a H3R26Cit-H3K27me complex. This new finding suggests that detection of H3K27me may be helpful to recognize H3R26Cit indirectly.

Previously, most studies on histone modifications focused on acetylation, methylation, and phosphorylation. The studies of histone citrullination are limited, especially for gastric cancer. Protein citrullination, also known as deamination, refers to a post-translational modification of arginine to citrulline (20, 21). Studies on the relationship between histone citrullination and tumors have mainly focused on histone H3. Thalin and coworkers reported that elevated H3Cit in peripheral blood predicted poor prognosis for advanced cancer patients including colorectal cancer, gastric cancer, and breast cancer (10). Neutrophil extracellular traps (NETs) could be a source of citrullinated histones in the blood. PADI4 mediates histone citrullination in NETs formation (11, 22, 23). Protein citrullination also participates in the regulation of stem cell pluripotency, cancer-related genes, and immune responses (24–27). Although we characterized the antigen labeled by IPO-38 antibody, the exact clinical significance of citrullinated histone 3 needs further investigation.

The protein citrullination refers to a chemical conversion of arginine to citrulline, which is catalyzed by peptidylarginine deiminases (PADIs) in human beings (28). Among PADIs family, PADI4 carries a nuclear localization signal, and is mainly located in the nucleus (29). PADI2 might also undergo nuclear translocation in some cells to modify histones (26). Since both PADI4 and PADI2 might be involved in the citrullination of histones, we examined the expression levels of H3R26Cit, PADI4, and PADI2 synchronously and confirmed that PADI4, but not PADI2, regulates H3R26Cit formation. In addition, we found that the expression levels of mRNA and protein of PADI2 and PADI4 was inconsistent, which might be attributed to post-transcriptional modification of mRNA or post-translational modification of protein (30, 31).

In addition to intracellular histone citrullination, PADI4 in neutrophils can facilitate histone citrullination of NETs. This kind of extracellular histone modification facilitated ovarian cancer premetastatic niche formation in the omentum. Interfering NETs formation could inhibit cancer metastasis (32). Yuzhalin and colleagues indicated that extracellular histone modifications can promote liver metastasis of colorectal cancer (12). Therefore, protein citrullination of the extracellular matrix and microenvironment may play an important role on tumor progression. Higher levels of PADI4 have been reported in peripheral blood in several types of cancers (33).

Histone modification is a complex area. The precise correlation of H3R26Cit and H3K27me3 or H3K27ac is largely unknown. In this paper, we identified the crosstalk between H3R26Cit and H3K27me3, which was mentioned by other study before (34). According to our results, the binding ability of IPO-38 antibody to antigen might be affected by their crosstalk, but more experiments need to be done. EZH2 is an enzyme that mediates methylation of H3K27me3 (34). EZH2 was found up-regulated in melanoma, lymphoma, breast cancer, and prostate cancer, and related to promoting tumorigenesis, cell proliferation, and epithelial mesenchymal transition (35). KDM6A and KDM6B are enzymes involved in demethylation of H3K27me3 (36). Although we found that overexpression of PADI4 influences the expression levels of H3K27me3 and H3R26Cit, we did not find a confirm correlation between expression of PADI4 and methylation-related enzymes such as EZH2, KDM6A, and KDM6B. Our study clarified that PADI4 is a main regulatory enzyme of histone citrullination, at least in gastric cancer. This discovery will be used to optimize the sensitivity and specificity of IPO-38 as a diagnostic reagent for gastric cancer.

Since the technical limitations, we did not analyze the clinical correlations. Next, we prepare to immunize mice with synthetic histone-modified polypeptide antigen to obtain specific monoclonal antibody, and then perform immunohistochemistry by new developed specific monoclonal antibody. We will establish a sandwich ELISA reagent to examine blood samples from patients.

## Author Contributions

SS and YY formulated the experimental concept and design. ZX, JL, JJ, and RY performed experiments. ZZ and YY supported the research. All authors wrote, reviewed, and revised the manuscript.

### Conflict of Interest Statement

The authors declare that the research was conducted in the absence of any commercial or financial relationships that could be construed as a potential conflict of interest.
